# Supplementary material for: Colossal and reversible barocaloric effect in liquid-solid-transition materials n-alkanes
Source: Nat Commun. 2022 Feb 1;13:596. doi: 10.1038/s41467-022-28229-4 (PMC8807803; doi:10.1038/s41467-022-28229-4)
Supplement: Supplementary file 1 — Supplementary Information [file 41467_2022_28229_MOESM1_ESM.pdf]

Supplementary Information

Colossal and Reversible Barocaloric Effect in Liquid-Solid-Transition Materials *n*-alkanes

J. C. Lin *et al.*

Supplementary Note 1. Why the pressure-driven isothermal entropy change ΔS calculated in this paper was underestimated?

At present, the quasi-direct method is still the most commonly used way to evaluate the isothermal entropy change ($\Delta S(p,T) = S_t(p,T) - S(p_{atom},T)$), and the key is to obtain the total entropy under different pressures, i.e. $S_t(p,T)$. Usually, $S_t(p,T)$ can be calculated through the following relation:

$$S_t(T,p) = \begin{cases} S(T_0,p) + \int_{T_0}^{T} \frac{c_L(p,T')}{T'} dT' & T_0 \le T \le T_1 \\ S(T_1,p) + \int_{T_1}^{T} \frac{1}{T'}\left(c_M(p,T') + \left(\frac{dQ(p,T')}{dt}\right)\left(\frac{dT'}{dt}\right)^{-1} \right) dT & T_1 \le T \le T_2 \\ S(T_2,p) + \int_{T_2}^{T} \frac{c_H(p,T')}{T'} dT & T > T_2 \end{cases}, \tag{S1}$$

where T_1 and T_2 are the start and end temperatures of the phase transition, $\frac{dQ(p,T')}{dt}$ is the heat flux, $\frac{dT}{dt}$ is temperature sweep rate, and $S(T_0,p)$ can also be written as $\int_{T=0\text{K}}^{T_0} \frac{c_L(p_{\text{atm}},T)}{T} dT - \int_{p_{\text{atm}}}^{p} \left(\frac{\partial V(p',T)}{\partial T}\right)_{p'} dp'$.

Otherwise, parameters including $C(p,T)$ and $V(p,T)$ are hardly available in the literature and their measurements are also challenging. Few previous studies have constructed $S_t(p,T)$ based on experimentally measured $C(p,T)$ and $V(p,T)$ curves. Therefore, how did previous studies calculate the isothermal entropy change?

1) The reported strategies for calculating isothermal entropy change:

Strategy 1: $\Delta S(p,T)$ is calculated based on the entropy change from phase transition. Therefore, we could calculate $\Delta S(p,T)$ only by using the heat flux curve under different pressures according to the following formula,

$$\Delta S(p,T) = \int_{T_0}^{T} \frac{1}{T'}\left(\frac{dQ(p,T')}{dt}\right)\left(\frac{dT'}{dt}\right)^{-1} dT - \int_{T_0}^{T} \frac{1}{T'}\left(\frac{dQ(p_{atm},T')}{dt}\right)\left(\frac{dT'}{dt}\right)^{-1} dT, \tag{S2}$$

which was applied in the BC study including Ni-Mn-In[1], La-Fe-Si-Co[2], Mn_3GaN[3], NPG[4] and so on.

Strategy 2: $\Delta S(p,T)$ is calculated by combining the entropy change from the phase transition and "additional" entropy change $\Delta S_+(p)$ where the assumption $\frac{\partial V(p,T)}{\partial T} \simeq \frac{\partial V(p_{\text{atm}},T)}{\partial T}$ was adopted.

For cases of ammonium sulphate[5], AgI[6], [TPrA][Mn(dca)$_3$][7], and NPG[8], the "additional" entropy change in the temperature range away from the phase transition can be written as

$$\Delta S_+(p) = -\frac{1}{m}\int_{p_{atm}}^{p}\left(\frac{\partial V(p',T)}{\partial T}\right)_{p'} dp' \tag{S3}$$

according to the Maxwell relation $-\frac{1}{m}\left(\frac{\partial V}{\partial T}\right)_p = \left(\frac{\partial S}{\partial p}\right)_T$. Under this strategy, the assumption $\frac{\partial V(p,T)}{\partial T} \simeq \frac{\partial V(p_{atm},T)}{\partial T}$ was adopted, meaning that the changes in pressure do not affect the evolution of volume with temperature. So, Eq. (S3) can be written as:

$$\Delta S_+(p) = -\frac{1}{m}\int_{p_{atm}}^{p}\left(\frac{\partial V(p',T)}{\partial T}\right)_{p'} dp' \simeq -\frac{1}{m}\left(\frac{\partial V(p_{atm},T)}{\partial T}\right)(p - p_{atm}). \tag{S4}$$

For the other cases such as layered hybrid perovskite[9], and C60[10], the "additional" entropy change was calculated based on Eq. (S3) in the temperature range below the reference temperature T_0 (which is lower than T_1), and

$$\Delta S_+(p) = \int_{T_2}^{T}\frac{c_H(p,T')}{T'}dT - \int_{T_2}^{T}\frac{c_H(p_{atm},T')}{T'}dT \tag{S5}$$

in the temperature range above T_2. When constructing the heat capacity under pressure (i.e., $C_H(p,T')$), the assumption $\frac{\partial V(p,T)}{\partial T} \simeq \frac{\partial V(p_{atm},T)}{\partial T}$ was also adopted.

2) Our comments on the above strategies:

To obtain a more accurate isothermal entropy change, **Strategy 2** considers the additional entropy change $\Delta S_+(p)$, where the assumption $\frac{\partial V(p,T)}{\partial T} \simeq \frac{\partial V(p_{atm},T)}{\partial T}$ was adopted. For a material with a high elastic modulus, the above assumption is basically applicable and will not lead to a large error in $\Delta S_+(p)$. In this case, **Strategy 2** gives a more accurate isothermal entropy than **Strategy 1** does. Otherwise, in regard to organic compounds or the liquids, the materials show a high compressibility and $\frac{\partial V(p,T)}{\partial T}$ will change significantly as pressure increases. For example, the relation of $\left(\frac{\partial V(p_{atm},T)}{\partial T}\right) \simeq 2.04\left(\frac{\partial V(p_{150MPa},T)}{\partial T}\right)$ for liquid $C_{18}H_{18}$[11] and $\left(\frac{\partial V(p_{atm},T)}{\partial T}\right) \simeq 2.35\left(\frac{\partial V(p_{173MPa},T)}{\partial T}\right)$ for the acetoxy silicon rubber[12] can be calculated from previous studies. So, if the above assumption is still used in organic material, $|\Delta S_+(p)|$ will be seriously overestimated according to Eq. (S4). Furthermore, the greater the applied pressure is, the greater the overestimation.

From the above discussions, we can see that the previous work also applied **Strategy 2** to organic compounds[8,13] as the experimentally measured $C(p,T)$ and $V(p,T)$ curves were unavailable.

Inevitably, it led to a severe overestimation of isothermal entropy change. This is why Li et al calculated the isothermal entropy change of NPG based on **Strategy 1**[4]. In other words, they solely took into account the entropy change from the phase-transition and ignored the contribution beyond the phase transition.

3) Determination of isothermal entropy change for *n*-alkanes.

In our cases, only V(*p,T*) data for liquid $C_{18}H_{38}$ and $C_{16}H_{34}$ are available, but V(*p,T*) for the solid state and C(*p,T*) are still unavailable. Meanwhile, $\left(\frac{\partial V(p_{atm},T)}{\partial T}\right) \simeq 2.04\left(\frac{\partial V(p_{150MPa},T)}{\partial T}\right)$ for liquid $C_{18}H_{18}$ indicates that **Strategy 2** is not suitable for *n*-alkanes. Therefore, similar to Li did[4], we calculated the isothermal entropy change of $C_{18}H_{38}$ and $C_{16}H_{34}$ based on **Strategy 1**.

For $C_{18}H_{38}$ and $C_{16}H_{34}$, the entropy changes of the liquid-solid transition decrease with increasing pressure as shown in Supplementary Fig. 5a, which mainly originates from the additional contribution (shown in Supplementary Fig. 5b) beyond the phase transition. The high-pressure Raman and MD simulation indicate that the same pressure can induce a more significant configuration change in liquid state than in solid state, resulting in a larger pressure-induced entropy decrease in the liquid state ($\left|\Delta S_{+}^{L}(p,T)\right|$) than that in the solid state ($\left|\Delta S_{+}^{S}(p,T)\right|$). Combining the phase transition contribution (Supplementary Fig. 5c) and the additional contribution, we constructed the real isothermal entropy change (Supplementary Fig. 5d). Obviously, **due to ignoring $\Delta S_{+}(p)$, the calculated isothermal entropy change reported in this paper (based on Strategy 1, only considering the contribution from the phase transition) is actually underestimated.**

Supplementary Note 2. About the distorted exothermic/endothermic peaks in the temperature-dependent T(*t*) curves.

For the present *n*-alkanes, applying pressure pushes the liquid-solid transition (T_{LS}) to higher temperatures. Therefore, lowering the temperature or applying pressure stabilizes the solid state, while raising the temperature or releasing pressure favors the liquid state.

At temperatures slightly higher than T_{LS}, applying pressure favors the solid state, and thus the liquid to solid transition is rapid. The corresponding exothermic peak is thus sharp and tall. However, although releasing pressure would help the solid state transfer back to the liquid state, the low temperature does favor the solid state. Thus, the solid to liquid transition during the pressure release process is not as sharp as the liquid to solid transition caused by pressure loading; accordingly, the endothermic peak is distorted into a short peak and a broad tail.

At temperatures much higher than T_{LS}, releasing pressure in the solid state is favorable to the liquid state, which gives rise to a rapid solid to liquid phase transition. In contrast, pressure loading fails to obtain a rapid liquid to solid transition since the liquid state is more stable than the solid state at high temperatures. As a result, the exothermic peak (corresponding to the liquid to solid transition) in T(*t*) is distorted (a small peak plus a long tail), while the endothermic peak (corresponding to the solid to liquid transition) is tall and sharp.

In the solid state (e.g., at 294 K for $C_{18}H_{38}$), pressure is not able to introduce any phase transitions, so both the endothermic and exothermic peaks in the T(*t*) curves have a single peak with similar shapes. Only at high pressures, e.g., at 500 MPa, the endothermic peak is slightly sharper than the exothermic peak because the pressure release process is more rapid than the pressure application process.

Since the testing apparatus cannot be fully insulated, a long exothermic/endothermic time will significantly lower the adiabatic temperature change, $|\Delta T_d|$. For the sake of accuracy, here the measured $|\Delta T_d|$ value will be ignored when the exothermic/endothermic time takes more than 60 s. Additionally, the rate of pressure change, which determines how fast the pressure is changed, will also affect the $|\Delta T_d|$ value. In our experiment, the pressure change is manually operated, and thus the pressure-releasing process is more rapid than the pressure-applying process. Therefore, in Fig. 1c and Fig. 1d, $|\Delta T_d|$ values corresponding to the pressure-applying process were ignored if the samples lie in the solid state or in the liquid state but the applied pressure is lower than the critical value.

Supplementary Note 3. The details of the theoretical calculation.

To gain insight into the microprocess of solid-liquid phase change and the thermodynamic parameters, we perform classic molecular dynamics (MD) simulations using the LAMMPS package with periodic boundary conditions[14]. The adaptive intermolecular reactive empirical bond order (AIREBO) potential for a system of C-H atoms was used in our MD simulations[15]. The Newton equation of motion was integrated by the velocity Verlet algorithm and the time-step was 0.5 fs. We used the coexisting solid-liquid phases to simulate the melting temperature of $C_{18}H_{38}$, since the two-phase method is more accurate for melting simulation[16,17]. To construct the coexisting solid-liquid phases, we first performed the MD simulations started with the 7×7×3 supercell (8232 atoms) in the NPT ensemble at 200 K to obtain the solid phase (1,000,000 time steps), and then heated the solid phase to 550 K (far beyond the melting point) to obtain the liquid phase (1,000,000 time steps). The obtained solid and liquid phases were constructed into the coexisting phase including solid-liquid interface. We performed the MD simulation for the coexisting solid-liquid phases in the NVE ensemble (1,000,000 time steps) to obtain the melting temperature. However, the two-phase method cannot obtain useful thermodynamic data of $C_{18}H_{38}$ from the solid to liquid phase during heating. We have to use the normal one-phase method, although it usually overestimates the melting temperature due to the superheating problem. Here, we performed MD simulations by directly heating the solid phase of the 7×7×3 supercell from 50 K to 550 K in the NPT ensemble for 15 ns (30,000,000 time steps). We evaluated the entropy change ΔS from the enthalpy change ΔH of solid-liquid phases by using $\Delta S = \Delta H/T_m$. Since the obtained temperature range of the phase change is not narrow, the theoretically estimated entropy change may be inaccurate to a certain extent. However, it would not hinder us from analyzing the law of the phase change under pressure compared with the experimental data. The radial distribution function and the dihedral-angle statistics use RINGS code from the MD results[18].

To investigate the lattice vibrational entropy of $C_{18}H_{38}$ in the solid phase, we first calculate the vibrational frequencies using the finite displacement method implemented in the Vienna Ab initio Simulation Package (VASP)[19] and the PHONOPY code[20]. A 2×2×2 supercell of a fully-relaxed $C_{18}H_{38}$ structure containing 448 atoms is constructed. The Brillouin zone (BZ) was sampled with an 8×8×2 *k*-point mesh for the structural relaxation of the unit-cell and a 5×5×1 *k*-point mesh for the supercell calculations. The exchange-correlation interaction was treated by the generalized gradient approximation (GGA), which is parameterized by Perdew-Burke-Ernzerhof (PBE)[21]. We

use the Grimme DFT-D3 method to describe the long-range van der Waals (VDW) interactions[22]. A cut-off energy of 500 eV was used for the plane-wave basis expansion. The lattice constants and ion positions are optimized using the Broyden-Fletcher-Goldfarb-Shanno (BFGS) quasi-Newton algorithm with a force convergence criterion of less than 10^{-3} eV/Å. The vibrational entropy is calculated using the equation:

$$S_{vib}=\frac{1}{2T}\sum_{q\nu}\hbar\omega(q\nu)\coth(\hbar\omega(q\nu)/2k_BT)-k_B\sum_{q\nu}\ln[2\sinh(\hbar\omega(q\nu)/2k_BT)], \quad (S6)$$

where ω is the vibrational frequency, *q* is the wave vector, and *v* is the index of the phonon mode[23].

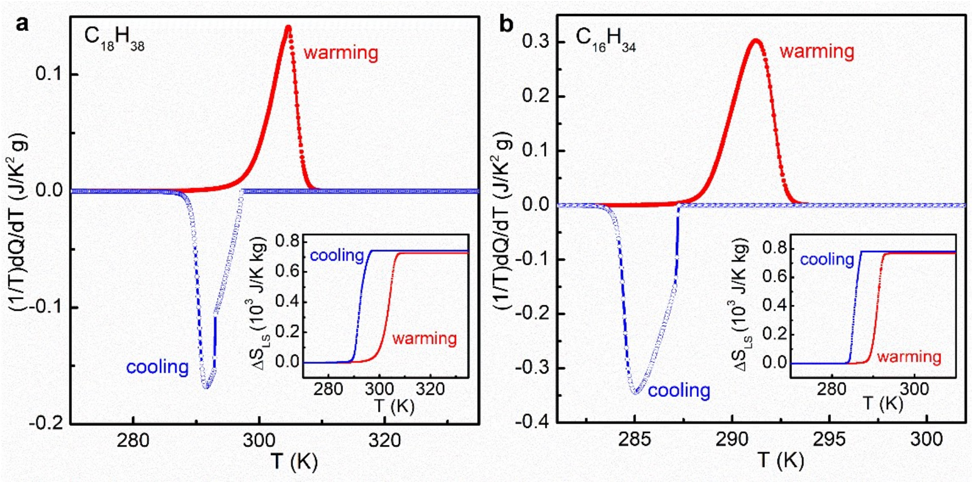

Supplementary Fig. 1 Calorimetric heat flow curves ((1/T) (dQ/dT)-T) for $C_{18}H_{38}$ and $C_{16}H_{34}$ after subtracting the baseline. **a** The (1/T) (dQ/dT)-T curves under ambient pressure for $C_{18}H_{38}$ measured in both heating and cooling runs. Inset: The entropy change ΔS_{LS} around the first-order phase transition derived from the heat flow curves. **b** The (1/T) (dQ/dT)-T curves for $C_{16}H_{34}$ measured on both heating and cooling runs. Inset: The entropy change ΔS_{LS} around the first-order phase transition derived from the heat flow curves.

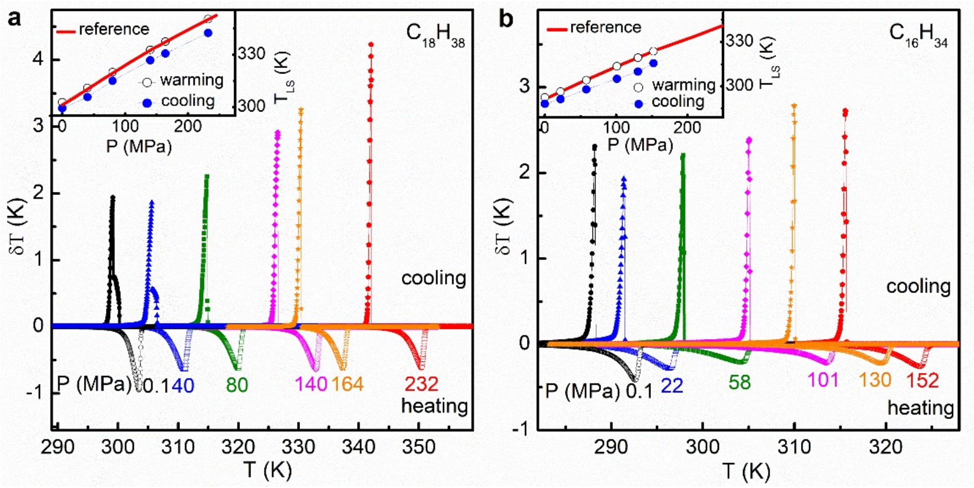

Supplementary Fig. 2 Temperature dependent DTA curves under different pressures for $C_{18}H_{38}$ and $C_{16}H_{34}$ after subtracting the baseline. **a** Temperature dependent DTA curves of $C_{18}H_{38}$ under different pressures measured during both heating and cooling runs. Inset: The peak temperature under different pressures, which was shown along with the reported values[24,25]. **b** Temperature dependent DTA curves of $C_{16}H_{34}$ under different pressures measured during both heating and cooling runs. Inset: The peak temperature under different pressures, which is shown along with the reported values.

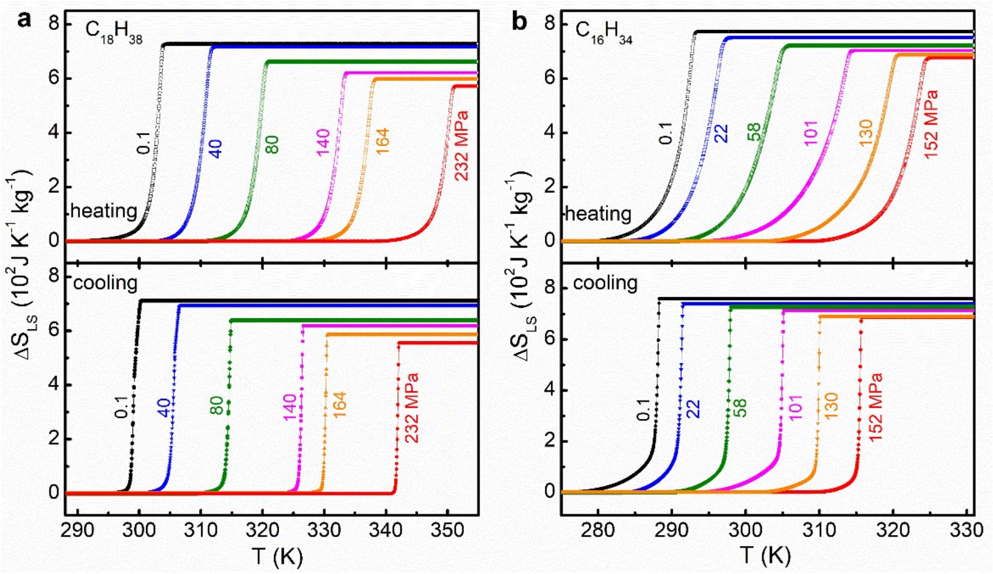

Supplementary Fig. 3 The entropy change curves $\Delta S_{LS}(T)$ purely due to the first-order liquid-solid transition calculated from the DTA curves. The $\Delta S_{LS}(T)$ curves under different pressures for $C_{18}H_{38}$ **a** and $C_{16}H_{34}$ **b**, which were constructed by combining the DSC results at ambient pressure and DTA signals recorded at different pressures.

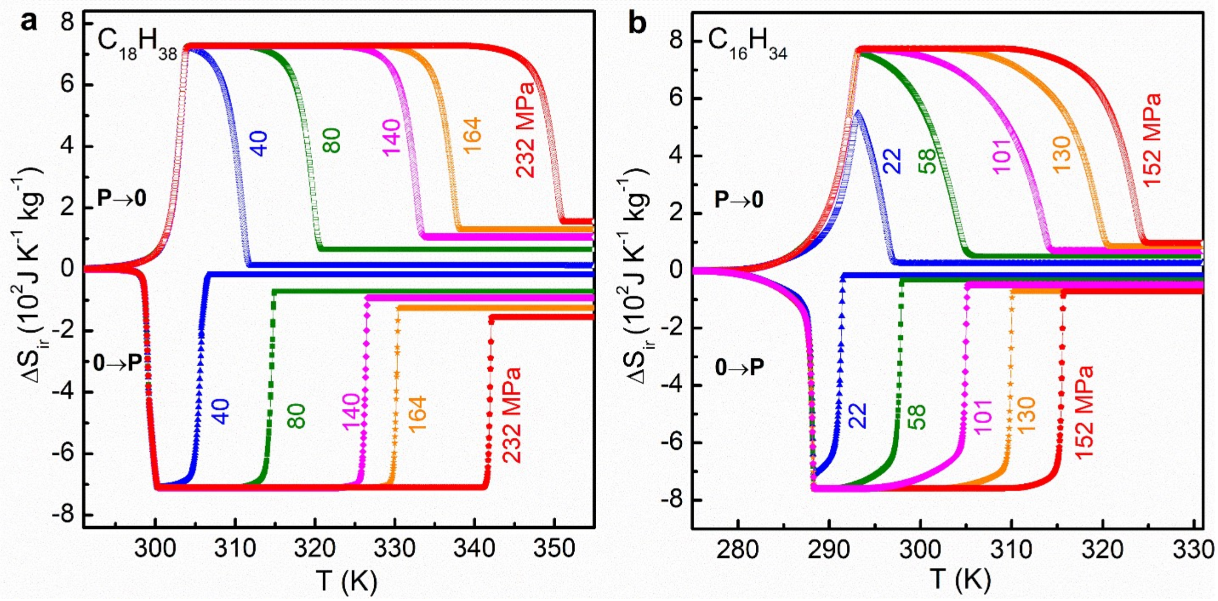

Supplementary Fig. 4 The irreversible entropy change under different pressures, $\Delta S_{ir}(T)$. $\Delta S_{ir}(T)$ curves under different pressures for $C_{18}H_{38}$ **a** and $C_{16}H_{34}$ **b**, which were derived from the calculated total entropy, $\Delta S_t(T)$. Based on $\Delta S_{ir}(T)$ curves, the reversible entropy change can be calculated. The specific details can be referred to Ref. 13.

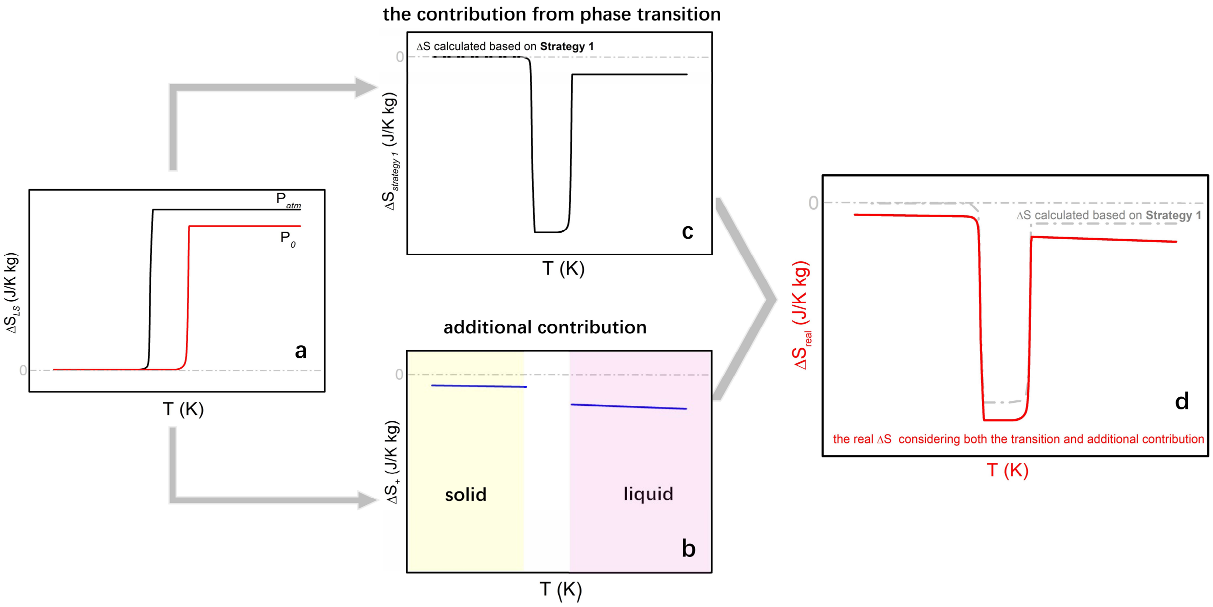

Supplementary Fig. 5 Schematic diagram of the pressure-induced isothermal entropy change before and after considering the additional contribution. **a** The entropy change only from the phase change under different pressures; **b** The pressure-induced additional entropy change beyond the phase transition; **c** the pressure-induced isothermal entropy change based on **Strategy 1** (which only considers the contribution from the phase transition); **d** the isothermal phase transition by combining the phase transition and additional contribution.

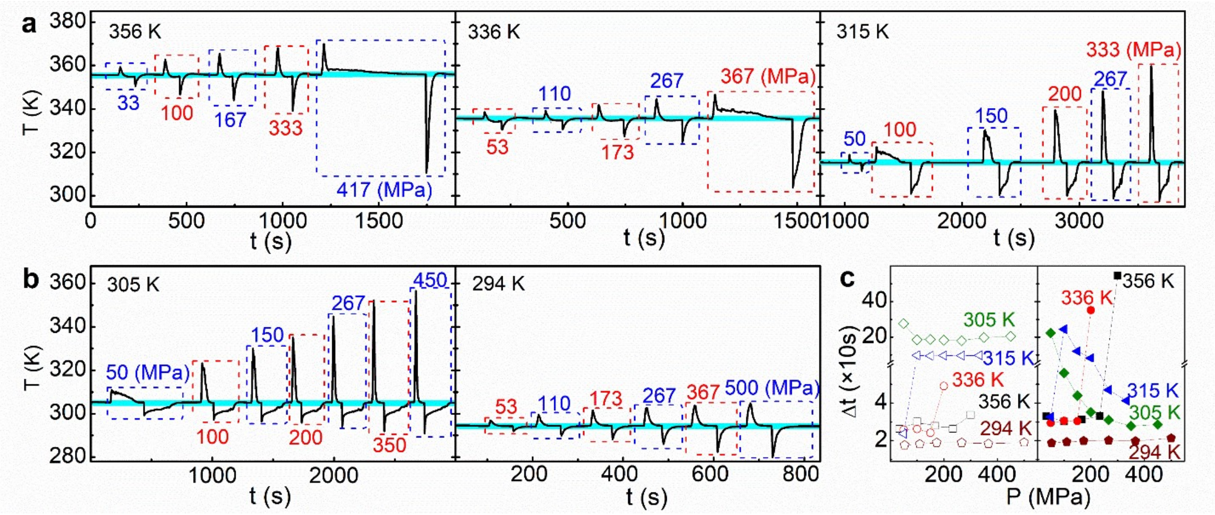

Supplementary Fig. 6 The adiabatic temperature change, ΔT_d, for $C_{18}H_{38}$ measured by the direct method. The ΔT_d values corresponding to different pressures applied at 356 K, 336 K and 315 K **a**, 305 K and 294 K **b**, respectively. **c** The time spent in the complete exothermic (corresponding to pressure-applying, right panel) and endothermic (corresponding to pressure-releasing, left panel) processes at different temperatures and pressures. Since the present testing apparatus cannot be fully insulated, the precision of $|\Delta T_d|$ will be strongly affected by the exothermic and endothermic times. Therefore, we mainly counted the $|\Delta T_d|$ value as $\Delta t < 60$ s.

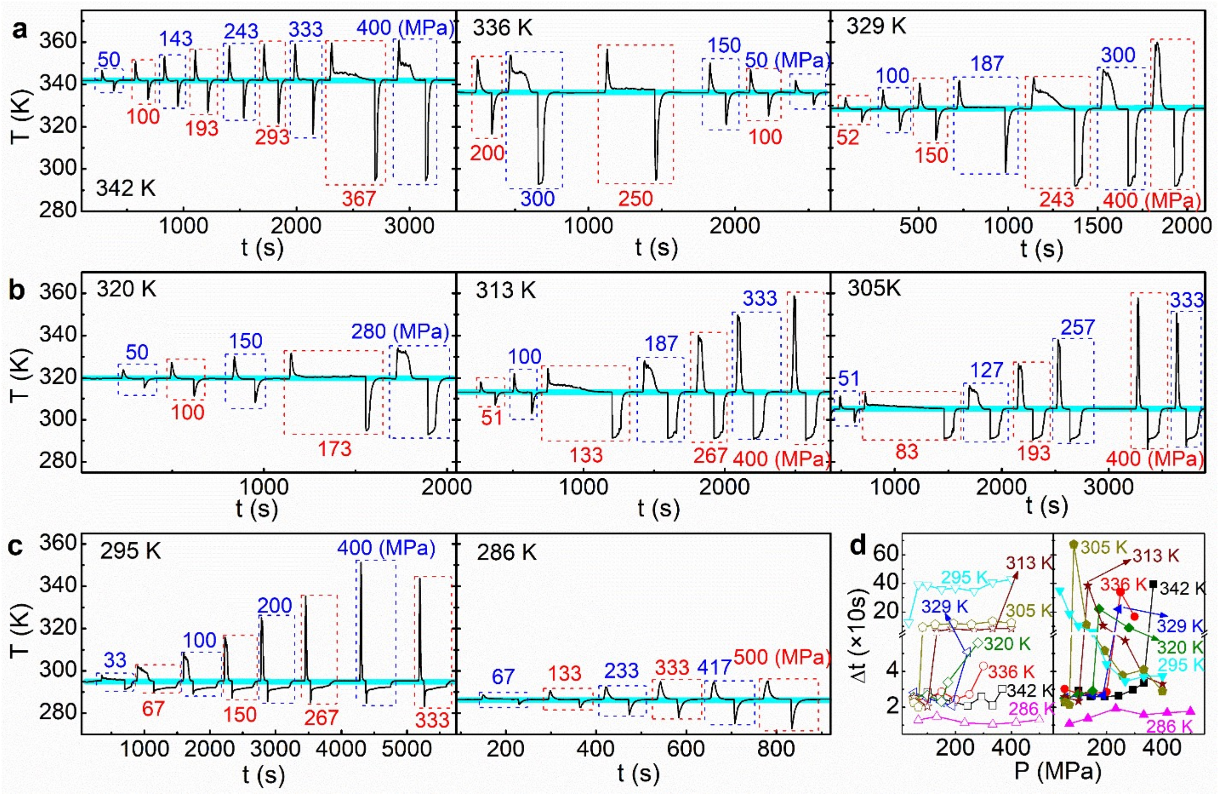

Supplementary Fig. 7 The adiabatic temperature change, ΔT_d, for $C_{16}H_{34}$ measured by the direct method. The ΔT_d values corresponding to different pressures applied at 342 K, 336 K and 329 K **a**, 320 K, 313 K and 305 K **b**, and 295 K and 286 K **c**. **d** The time spent in the complete exothermic (corresponding to pressure-applying, right panel) and endothermic (corresponding to pressure-releasing, left panel) processes at different temperatures and pressures. Since the present testing apparatus cannot be fully insulated, the precision of $|\Delta T_d|$ will be strongly affected by the exothermic and endothermic time. Therefore, we mainly counted the $|\Delta T_d|$ value as $\Delta t < 60$ s.

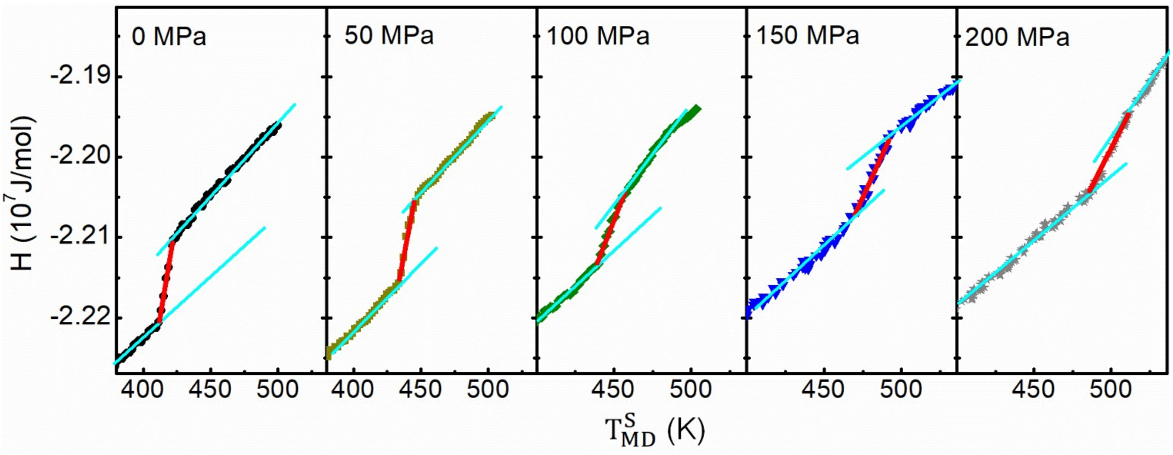

Supplementary Fig. 8 The enthalpy change at different pressures for $C_{18}H_{38}$ calculated by the normal one-phase MD methods.

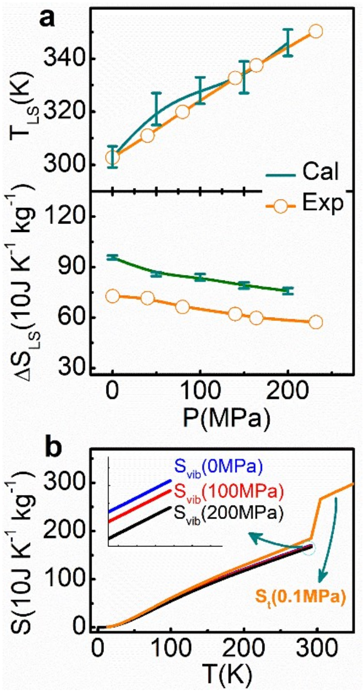

Supplementary Fig. 9 The theoretical calculation of thermodynamic information under different pressures for $C_{18}H_{38}$. **a** The LST temperature (T_{LS}) and entropy change (ΔS_{LS}) solely due to the L-S-T under different pressures determined from both the experiment and theoretical calculation; **b** The temperature dependent lattice vibration entropy (ΔS_{vib}) calculated by the DFT and the total entropy based on the experimental results.

As shown in the above picture, the evolutions of T_{LS} and ΔS_{LS} (calculated from the enthalpy change shown in Supplementary Fig. 8) with pressure present a high consistency with the experimental results. This indicates the rationality of the relevant models and parameters adopted in the present MD simulation. Previous studies definitely indicate that DFT is highly reliable in estimating the lattice vibration entropy (S_{vib}) of crystalline materials[23]. Therefore, the S_{vib} of $C_{18}H_{38}$ in the solid state was estimated by this mean and compared with the total entropy (S_t) obtained by experiments. The results indicate the S_{vib} always contributes dominantly to S_t for solid $C_{18}H_{38}$, while its proportion decreases as the temperature is raised. At 100 K, S_{vib} accounts for ~94.6% of S_t. When the temperature is close to T_{LS}, i.e., 290 K, the value drops to 92.3%, indicating the involvement of configuration contribution. When the pressure was increased from ambient pressure to 200 MPa, the S_{vib} was less affected.

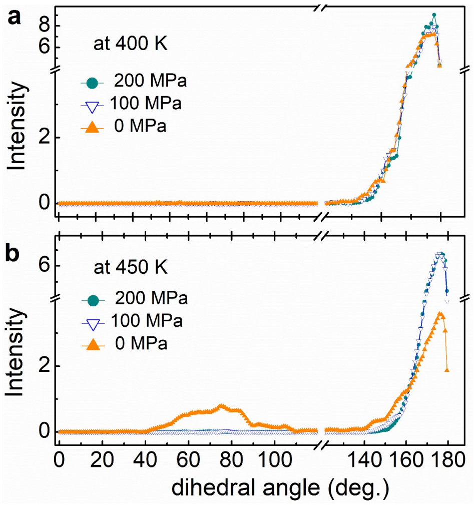

Supplementary Fig. 10 The distribution of dihedral angle between C-C bonds for $C_{18}H_{38}$ calculated by the MD simulation at different temperatures and pressures.

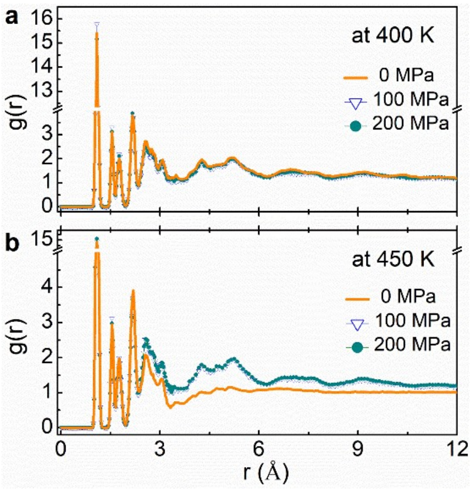

Supplementary Fig. 11 The theoretically obtained radial distribution function at different temperatures and pressures for $C_{18}H_{38}$.

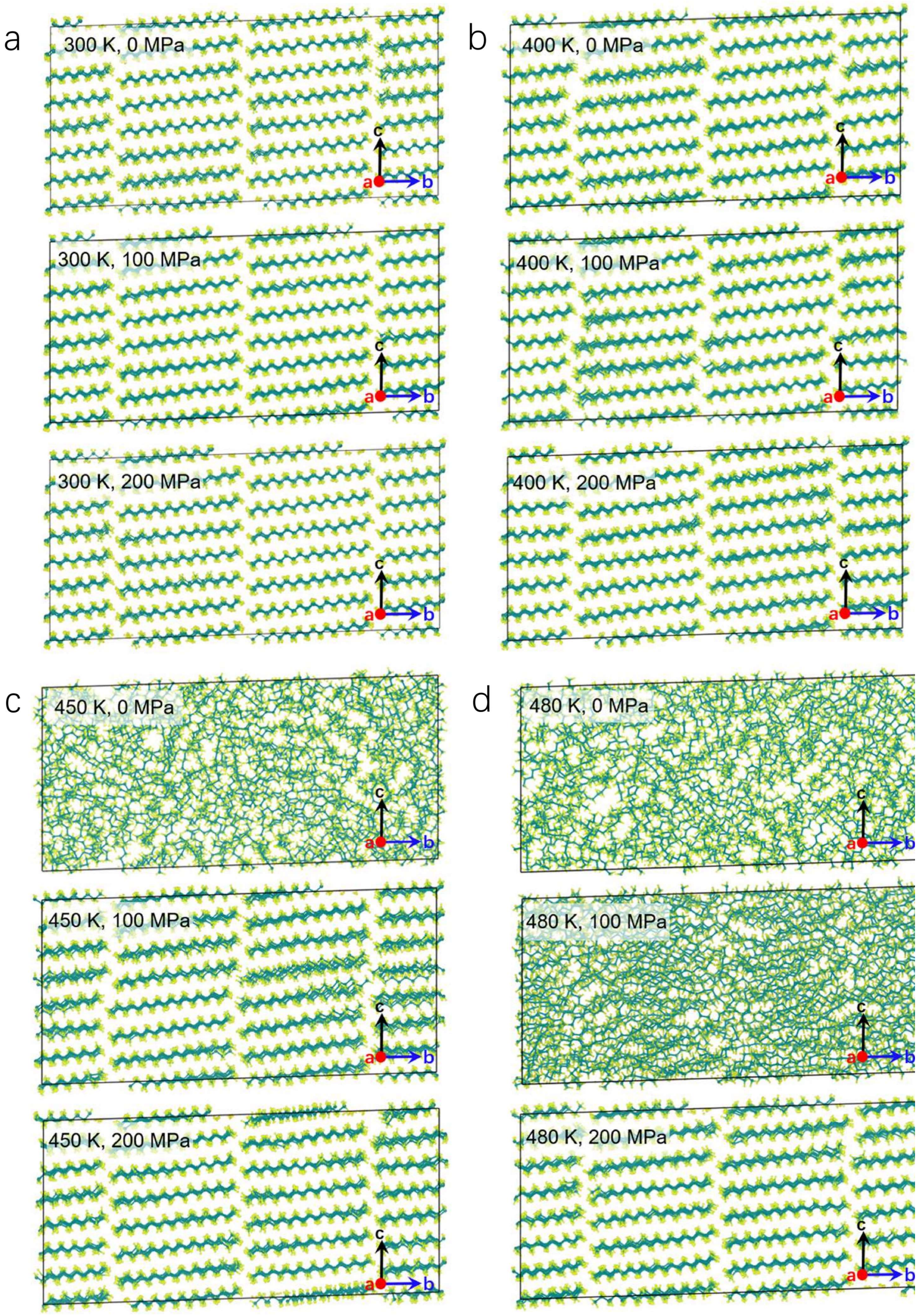

Supplementary Fig. 12 Theoretically calculated structures at different temperatures and pressures for $C_{18}H_{38}$.

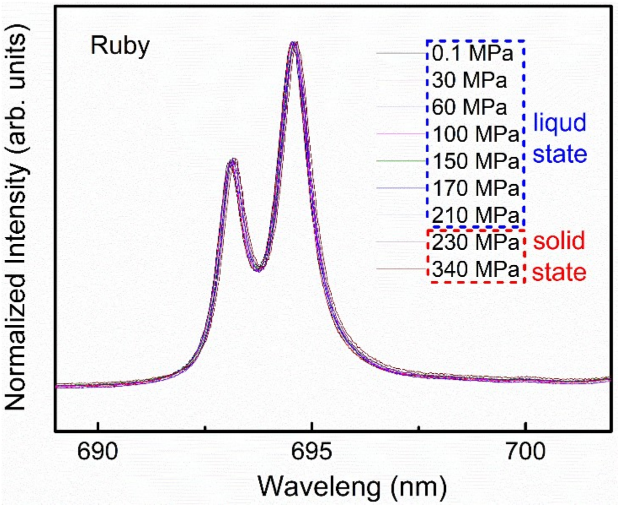

Supplementary Fig. 13 Ruby fluorescence spectrum under different pressures at 350 K. Ruby along with n-octadecane are sealed in the diamond pressure package. High-temperature conditions were obtained using a specially designed cryostat with the temperature error less than 0.5 K. The pressure was determined using the ruby fluorescence method considering the temperature correction.

As shown in Supplementary Fig. 13, the peak shape of the ruby fluorescence spectrum used for pressure calibration in high-pressure Raman measurement remains undistorted when the pressure drives the *n*-alkanes from liquid to solid (soft material), which means that hydrostatic pressure can be formed inside the sample when the pressure is applied to both states of *n*-alkanes[26]. Therefore, when *n*-alkanes are used for BC refrigeration, there is no need to add an additional liquid pressure transfer medium.

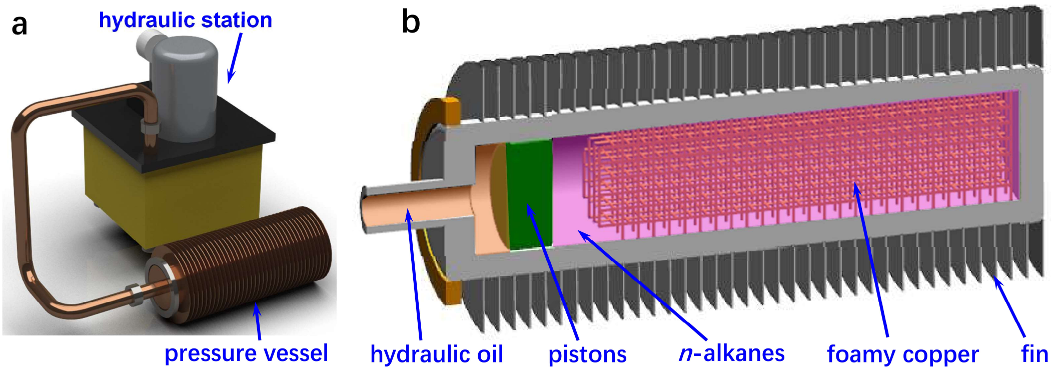

Supplementary Fig. 14 Profile **a** and sectional view **b** of the pressure vessel used in the BC refrigerator. In this strategy, the *n*-alkanes themselves can act as both the refrigerant and pressure transfer medium. The foamy copper is embedded into the pressure vessel to improve the heat exchange between the *n*-alkanes and the heat transfer medium. In future BC applications, the pressure vessel will be placed in the heat exchanger, and the fins arranged on its outer surface can accelerate the heat exchange with the heat transfer fluid.

Supplementary References

1. Mañosa, L., González-Alonso, D., Planes, A., Bonnot, E., Barrio, M., Tamarit, J. -L., Aksoy, S. & Acet, M. Giant Solid-state barocaloric effect in the Ni-Mn-In magnetic shape-memory alloy. *Nat. Mater.* **9**, 478-481 (2010).
2. Mañosa, L., González-Alonso, D., Planes, A., Barrio, M., Tamarit, J. -L., Titov, I. S., Acet, M., Bhattacharyya, A. & Majumdar, S. Inverse barocaloric effect in the giant magnetocaloric La-Fe-Si-Co compound. *Nat. Commun.* **2**, 595 (2011).
3. Matsunami, D., Fujita, A., Takenaka, K. & Kano, M. Giant barocaloric effect enhanced by the frustration of the antiferromagnetic phase in Mn_3GaN. *Nat. Mater.* **14**, 73-78 (2015).
4. Li, B., Kawakita, Y., Ohira-Kawamura, S., Sugahara, T., Wang, H., Wang, J. F., Chen, Y. N., Kawaguchi, S. I., Kawaguchi, S., Ohara, K., Li, K., Yu, D. H., Mole, R., Hattori, T., Kikuchi, T., Yano, S., Zhang, Z., Zhang, Z., Ren, W. J., Lin, S. C., Sakata, O., Nakajima, K. & Zhang, Z. D. Colossal barocaloric effects in plastic crystals. *Nature* **567**, 506-510 (2019).
5. Lloveras, P., Stern-Taulats, E., Barrio, M., Tamarit, J. -L., Crossley, S., Li, W., Pomjakushin, V., Planes, A., Mañosa, L., Mathur, N. D. & Moya, X. Giant barocaloric effects at low pressure in ferrielectric ammonium sulphate. *Nat. Commun.* **6**, 8801 (2015).
6. Aznar, A., Lloveras, P., Romanini, M., Barrio, M., Tamarit, J. -L., Cazorla, C., Errandonea, D., Mathur, N. D., Planes, A., Moya, X. & Mañosa, L. Giant barocaloric effects over a wide temperature range in superionic conductor AgI. *Nat. Commun.* **8**, 1851 (2017).
7. Bermúdez-García, J. M., Sánchez-Andújar, M., Castro-García, S., López-Beceiro, J., Artiaga, R. & Senarís-Rodríguez, M. A. Giant barocaloric effect in the ferroic organic-inorganic hybrid $[TPrA][Mn(dca)_3]$ perovskite under easily accessible pressures. *Nat. Commun.* **8**, 15715 (2017).
8. Lloveras, P., Aznar, A., Barrio, M., Negrier, P., Popescu, C., Planes, A., Mañosa, L., Stern-Taulats, E., Avramenko, A., Mathur, N. D., Moya, X. & Tamarit, J. -L. Colossal barocaloric effects near room temperature in plastic crystals of neopentylglycol. *Nat. Commun.* **10**, 1803 (2019).
9. Li, J. N., Barrio, M., Dunstan, D. J., Dixey, R., Lou, X. J., Tamarit, J. -L., Phillips, A. E. & Lloveras, P. Colossal reversible barocaloric effects in layered hybrid perovskite $(C_{10}H_{21}NH_3)_2MnCl_4$ under low pressure near room temperature. Adv. Funct. Mater. **31**, 2105154 (2021).

10. Li, J. N., Dunstan, D., Lou, X. J., Planes, A., Mañosa, L., Barrio, M., Tamarit, J. -L. & Lloveras, P. Reversible barocaloric effects over a large temperature span in fullerite C_{60}. *J. Mater. Chem. A* **8**, 20354-20362 (2020).
11. Dutour, S., Daridon, J. L. & Lagourette, B. Pressure and temperature dependence of the speed of sound and related properties in normal octadecane and nonadecane. *Int. J. Thermophys.* **21**, 173-184 (2000).
12. Imamura, W., Usuda, É. O., Paixão, L. S., Bom, N. M., Gomes, A. M. & Carvalho, A. M. G. Supergiant barocaloric effects in acetoxy silicone rubber over a wide temperature range: great potential for solid-state cooling. *Chinese J. Polym. Sci.* **38**, 999-1005 (2020).
13. Aznar, A., Lloveras, P., Barrio, M., Negrier, P., Planes, A., Mañosa, L., Mathur, N. D., Moya, X. & Tamarit, J. -L. Reversible and irreversible colossal barocaloric effects in plastic crystals. *J. Mater. Chem. A* **8**, 639-647 (2020).
14. Plimpton, S. Fast parallel algorithms for short-range molecular dynamics. *J. Comput. Phys.* **117**, 1-19 (1995).
15. Stuart, S. J., Tutein, A. B. & Harrison, J. A. A reactive potential for hydrocarbons with intermolecular interactions. *J. Chem. Phys.* **112**, 6472-6486 (2000).
16. Yoo, S., Zeng, X. C. & Morris, J. R. The melting lines of model silicon calculated from coexisting solid–liquid phases. *J. Chem. Phys.* **120**, 1654-1656 (2004).
17. Marbeuf, A. & Brown, R. Molecular dynamics in *n*-alkanes: Premelting phenomenon and rotator phases. *J. Chem. Phys.* **124**, 054901 (2006).
18. Roux, S. L. & Jund, P. Ring statistics analysis of topological networks: New approach and application to amorphous GeS_2 and SiO_2 systems. *Comp. Mat. Sci.* **49**, 70-83 (2010).
19. Kresse, G. and Joubert, D. From ultrasoft pseudopotentials to the projector augmented-wave method. *Phys. Rev. B* **59**, 1758-1775 (1999).
20. Togo, A. and Tanaka, I. First principles phonon calculations in materials science. *Scr. Mater.* **108**, 1-5 (2015).
21. Perdew, J. P., Burke, K. & Ernzerhof, M. Generalized gradient approximation made simple. *Phys. Rev. Lett.* **77**, 3865-3868 (1996).
22. Grimme, S., Antony, J., Ehrlich, S. & Krieg, H. A consistent and accurate *ab initio* parametrization of density functional dispersion correction (DFT-D) for the 94 elements H-Pu. *J. Chem. Phys.* **132**, 154104 (2010).

23. Wang, Y. L., Zhang, Y. S. and Wolverton, C. First-principles studies of phase stability and crystal structures in Li-Zn mixed-metal borohydrides. *Phys. Rev. B* **88**, 024119 (2013).
24. Nelson, R. R., Webb, W. & Dixon, J. A. First-order phase transition of six normal paraffins at elevated pressures. *J. Chem. Phys.* **33**, 1756-1764 (1960).
25. Baled, H. O., Xing, D., Katz, H., Tapriyal, D., Gamwo, I. K., Soong, Y., Bamgbade, B. A., Wu, Y., Liu, K., Mchugh, M. A. & Enick, R. M. Viscosity of n-hexadecane, n-octadecane and n-eicosane at pressures up to 243MPa and temperature up to 534K. *J. Chem. Thermodyn.* **72**, 108-116 (2014).
26. Takekiyo, T., Koyama, Y., Matsuishi, K. & Yoshimura, Y. High-Pressure Raman of n-Octane up to 15 GPa. *J. Phys. Chem. B* **124**, 11189-11196 (2020).
